# The *VDR* rs1544410 and rs11568820 Variants and the Risk of Osteoporosis in the Polish Population

**DOI:** 10.3390/ijms26020481

**Published:** 2025-01-08

**Authors:** Adam Kamiński, Anna Bogacz, Joanna Teresa Niezgoda-Nowak, Marta Podralska, Aleksandra Górska, Michał Soczawa, Bogusław Czerny

**Affiliations:** 1 Department of Orthopaedics and Traumatology, Independent Public Clinical Hospital No. 1, Pomeranian Medical University in Szczecin, Unii Lubelskiej 1, 71-252 Szczecin, Poland; emluc@wp.pl; 2 Department of Physiology, Poznan University of Medical Sciences, Święcickiego 6, 60-781 Poznan, Poland; 3 Department of Stem Cells and Regenerative Medicine, Institute of Natural Fibres and Medicinal Plants, Kolejowa 2, 62-064 Plewiska, Poland; marta.podralska@iwnirz.pl (M.P.); aleksandra.gorska@iwnirz.pl (A.G.); bczerny@wp.pl (B.C.); 4 Department of Pharmacology and Pharmacoeconomics, Pomeranian Medical University in Szczecin, 71-230 Szczecin, Poland; lek.joanna.niezgoda@gmail.com; 5 Department and Clinic of Urology and Urological Oncology, Pomeranian Medical University in Szczecin, al. Powstańców Wielkopolskich 72, 70-111 Szczecin, Poland; michal.soczawa@pum.edu.pl

**Keywords:** osteoporosis, genetic variants, *VDR1*, real-time PCR

## Abstract

Vitamin D affects bone metabolism and calcium-phosphate metabolism. Its deficiency leads to bone mineralization disorders and is the cause of abnormal skeletal development from fetal life to the period of completed skeletal growth. In later periods of life, vitamin D deficiency leads to bone metabolism disorders, i.e., osteoporosis. Disturbance of the balance between osteoblasts responsible for bone formation and osteoclasts associated with bone resorption results in reduced bone mass and bone weakening, and consequently leads to susceptibility to fractures. Analysis of genetic variants of the vitamin D receptor (*VDR*) concerns their relationship with metabolic bone diseases, and the results of previous studies assessing the relationship of polymorphisms with bone mineral density, fracture risk, or osteoporosis are not clear. Therefore, the aim of our study was to determine the effect of rs1544410 and rs11568820 polymorphisms of the *VDR* gene on the risk of developing osteoporosis in the Polish population. The study included 197 women with osteoporosis, 98 women with osteopenia, and 147 healthy controls. The real-time PCR method was used to determine the rs1544410 and rs11568820 polymorphisms of the *VDR1* gene. Analysis of the results in the group with osteopenia showed that for the rs1544410 polymorphism, heterozygous GA genotypes occurred in 37.8% of the study group and 47.6% of the controls (OR = 0.60; 95%CI: 0.34–1.05), and homozygous AA in 15.3% of the study group and 17.0% of the controls (OR = 0.68; 95%CI: 0.32–1.44) (*p* = 0.185, AIC = 332.4; AIC—Akaike information criterion). The best model for this variant turned out to be the dominant model OR = 0.62; 95%CI: 0.37–1.04; *p* = 0.071, AIC = 330.5. In the case of the rs11568820 polymorphism of the *VDR* gene, the GG genotype was more common in women with osteopenia compared to controls (75.5% vs. 70.1%). Genotypes containing at least one mutant A allele were present in 24.5% of women with osteopenia and 29.9% of controls (OR = 0.76; 95%CI: 0.43–1.36; *p* = 0.349; AIC = 332.9). Analyzing the rs1544410 polymorphism in women with osteoporosis, the GA genotype was present in 42.1% of the study group and 47.6% of patients with normal bone density (OR = 0.74; 95%CI: 0.46–1.19), and the AA genotype in 15.7% of the study group and 17.0% of controls (OR = 0.78; 95%CI: 0.41–1.46) (*p* = 0.441). In the case of the rs11568820 polymorphism, the GA genotype occurred in 22.3% of the study subjects and 27.2% of the control patients (OR = 0.76; 95%CI: 0.46–1.25), and the AA genotype in 2.0% of the study subjects and 2.7% of the controls (OR = 0.69; 95%CI: 0.17–2.83) (*p* = 0.511). For both variants, the model with the lowest AIC value was the dominant model, in which for the rs1544410 variant OR = 0.75; 95%CI: 0.48–1.17; *p* = 0.203; AIC = 472.0 was obtained, while for rs11568820—OR = 0.75; 95%CI: 0.47–1.22; *p* = 0.250; AIC = 472.3. The obtained results indicate that the rs1544410 and rs11568820 polymorphisms of the *VDR* gene do not affect the development of osteoporosis in the Polish population.

## 1. Introduction

Osteoporosis is considered a multifactorial, chronic metabolic disease of the skeletal system. It is characterized by a decrease in bone mineral density (BMD) and the occurrence of various bone microstructure disorders, which in turn contribute to numerous fractures in the skeletal system. An imbalance between osteoblasts responsible for bone formation, and osteoclasts associated with bone resorption, results in reduced bone mass and bone weakening, and consequently leads to susceptibility to fractures. Many factors influence the development of osteoporosis: age, gender, ethnicity, genetic predisposition, lifestyle (dietary and exercise habits), vitamin D deficiency, some chronic diseases, and medications taken [1,2,3,4].

The World Health Organization (WHO) has classified osteoporosis as a disease of civilization, due to its frequency of occurrence. It is estimated that osteoporosis affects more than 2 million people in Poland and more than 200 million people around the world. It occurs in older people and is more common in women during menopause, and 80% of cases in Poland are women [5,6]. Women during menopause are particularly susceptible to developing osteoporosis due to changes in the body, which involve the gradual cessation of ovarian activity, resulting in the cessation of menstruation and a decrease in estrogen levels. Decreased estrogen secretion promotes bone tissue metabolism disorders due to increased osteoclast activity and increased bone resorption [7].

It has been shown that genetic factors play a key role in the development of osteoporosis. Maintaining proper bone structure is controlled by many genes. The group of candidate genes that are most frequently studied include genes encoding type I collagen (*COL1A1*, *COL1A2*), estrogen receptor (ER), vitamin D receptor (*VDR*), as well as *OPG*, *LRP5,* and many others [8]. An important factor in the development of osteoporosis is vitamin D deficiency. The metabolically active form of vitamin D3 (1,25-dihydroxycholecalciferol) regulates calcium-phosphate metabolism and is responsible for bone tissue metabolism. Moreover, it determines the proper course of the differentiation, apoptosis, and proliferation of various cells of the immune system [9]. The vitamin D receptor gene is located on chromosome 12 (12q12-q14) and consists of nine coding exons [10]. Many studies have been conducted showing the correlation of *VDR* gene polymorphisms with the development of various diseases, including osteoporosis. The most frequently studied *VDR* gene polymorphisms include *Apa1* (rs7975232), *Bsm1* (rs1544410), *Cox2* (rs11568820), *Taq1* (rs731236), *Fok1* (rs10735810), which may affect gene expression and differential biological response to vitamin D [8,11]. It has been observed that *VDR* gene polymorphisms may interact with environmental factors such as vitamin D and calcium intake, as well as sun exposure, influencing the decrease in bone mineral density, which in turn is associated with an increased risk of bone fractures, leading to the development of osteoporosis. Identification of genetic variations in the *VDR* gene that are associated with low BMD would enable early detection of the risk of osteoporosis, which in turn would allow the implementation of appropriate prevention that could inhibit or delay the development of osteoporosis [12,13]. The literature data on the relationship between the *BsmI* and *Cdx2* polymorphisms of the *VDR* gene and the development of osteoporosis are ambiguous and sometimes contradictory, so it was decided to check their impact on the risk of developing osteoporosis in the Polish population.

The aim of our study was to examine the influence of *VDR* rs1544410 (G>A) and rs11568820 (G>A) polymorphisms on the risk of osteoporosis in Polish postmenopausal women. Analysis of *VDR* gene polymorphisms may help identify individuals at increased risk of developing osteoporosis and thus implement preventive measures in target risk groups.

## 2. Results

### 2.1. Genotype Analysis of the Studied Polymorphic Variants of the VDR Gene

The association between SNPs and the risk of reduced bone density was tested in different inheritance models (codominant, dominant, recessive, overdominant, and log-additive) using logistic regression. As shown in Table S1 for the rs1544410 variant in women with osteopenia, heterozygous GA genotypes occurred in 37.8% of the study group and 47.6% of the controls (OR = 0.60; 95%CI: 0.34–1.05), and homozygous AA in 15.3% of the study group and 17.0% of the controls (OR = 0.68; 95%CI: 0.32–1.44) (*p* = 0.185, AIC = 332.4). In the case of the second *VDR* gene variant (rs11568820), the GG genotype was more frequent in women with osteopenia (75.5% vs. 70.1% in the control group). Genotypes containing at least one mutant A allele were present in 24.5% of women with osteopenia and 29.9% of controls (OR = 0.76; 95%CI: 0.43–1.36; *p* = 0.349; AIC = 332.9).

Analysis of the studied *VDR* gene variants with the risk of osteoporosis showed no statistically significant differences (Table S2). However, similarly to osteopenia, genotypes containing the mutated A allele were more frequent in the control group. In the case of the rs1544410 variant in women with osteoporosis, the GA genotype occurred in 42.1% of the studied women and 47.6% of patients with normal bone density (OR = 0.74; 95%CI: 0.46–1.19). In contrast, the AA genotype was present in 15.7% of the studied women and 17.0% of the controls (OR = 0.78; 95%CI: 0.41–1.46) (*p* = 0.441). For the rs11568820 polymorphism, the GA genotype occurred in 22.3% of the study subjects and 27.2% of the control patients (OR = 0.76; 95%CI: 0.46–1.25), and the AA genotype in 2.0% of the study subjects and 2.7% of the controls (OR = 0.69; 95%CI: 0.17–2.83) (*p* = 0.511). For both variants, the model with the lowest AIC value was the dominant model, in which for the rs1544410 variant OR = 0.75; 95%CI: 0.48–1.17; *p* = 0.203; AIC = 472.0 was obtained, while for rs11568820 (OR = 0.75; 95%CI: 0.47–1.22; *p* = 0.250; AIC = 472.3).

### 2.2. Associations of VDR Gene Genotypes and Alleles with Densitometric Results

The next stage of statistical tests conducted in the selected groups was a comparative analysis of mean densitometric parameters depending on genotypes. No statistically significant differences were observed in any of the groups between the medians of BMD, T-score, and Z-score (Table 1).

**Figure 1:**
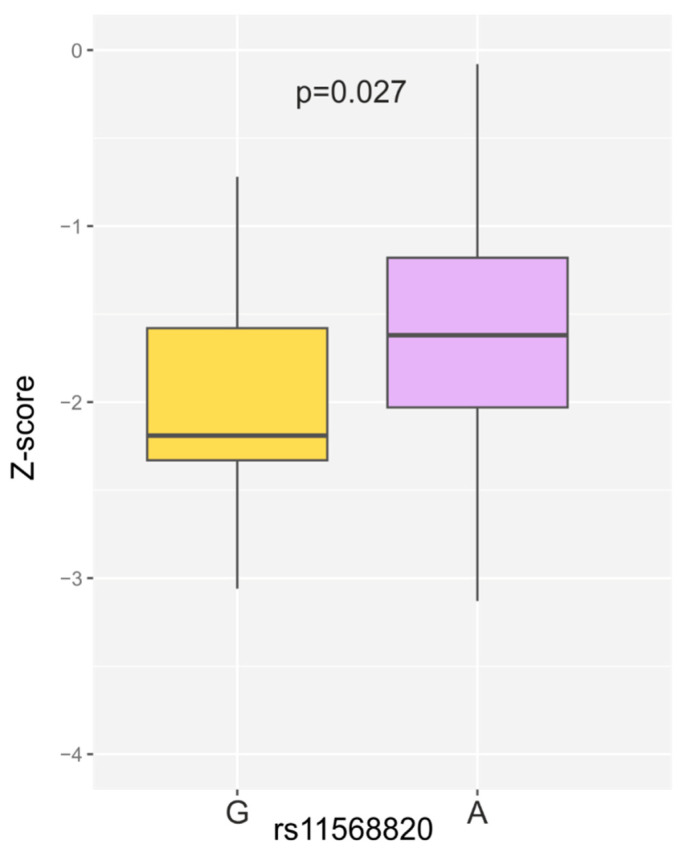
Box plot of Z-score values in patients with osteoporosis for the G and A alleles of the rs11568820 variant. Lines in the box plot indicate the median.

Analyzing the genotype distribution in women with osteoporosis, the medians for T−scores were GG: −3.71 (−3.80; −3.63), GA: −3.10 (−3.94; −2.72) and AA: −3.05 (−3.40; −2.71) (*p* = 0.232). A similar relationship was observed for Z−scores (GG: −2.47 (−2.68; −2.26), GA: −1.91 (−2.19; −1.53), AA: −1.62 (−2.03; −1.15); *p* = 0.120). Comparing the median Z−scores in patients with osteoporosis for alleles of the rs11568820 variant, the median for allele G was −2.19 (−2.33; −1.58), and for A −1.62 (−2.04; −1.17), *p* = 0.027 (Figure 1).

### 2.3. Association of Clinical Data with Analyzed VDR Gene Variants and Densitometric Results

A comparative analysis of the clinical parameters was performed, taking into account the division of the study group according to the T-score value with the genotypes of the analyzed *VDR* gene variants. The only statistically significant relationship was observed for the rs11568820 polymorphism. In the control group and with osteopenia, the last menstrual period occurred earlier in women with the AA genotype. In the osteopenia group, the mean age at last menstruation was 49.1 ± 4.1 in patients with the GG genotype, 50.4 ± 3.9 in GA, and 44.0 ± 5.3 in AA (*p* = 0.057), while in the control group it was 50.8 ± 4.2 in patients with the GG genotype, 50.2 ± 3.7 in heterozygotes, and 43.3 ± 2.1 in women with the AA genotype (*p* = 0.014). In patients with osteoporosis, the lowest mean age at last menstruation was also noted in women with the AA genotype (46.0 ± 8.5), but the difference was not statistically significant compared to the means obtained for the GG and GA genotypes (*p* = 0.716) (Table 2).

Correlations between densitometric parameters and age and BMI of patients in individual groups were also assessed. Correlations between age and BMI were positive in control and osteopenia groups (rho = 0.56; *p* < 0.001 for controls and rho = 0.34; *p* = 0.006 in osteopenia). The correlation of BMD%AM with age was also positive and statistically significant in all groups. There was also a positive statistically significant correlation between age and Z-score in the control and osteopenia groups (rho = 0.60; *p* = 0.003 for control and rho = 0.51; *p* < 0.001 in osteopenia). No statistically significant correlations were observed between densitometric results and the BMI of patients in the groups (Table S3).

### 2.4. Haplotype Analysis

The estimated frequency of haplotypes in the studied groups was presented in Table 3 using the HaploView 4.2 program.

**Figure 2:**
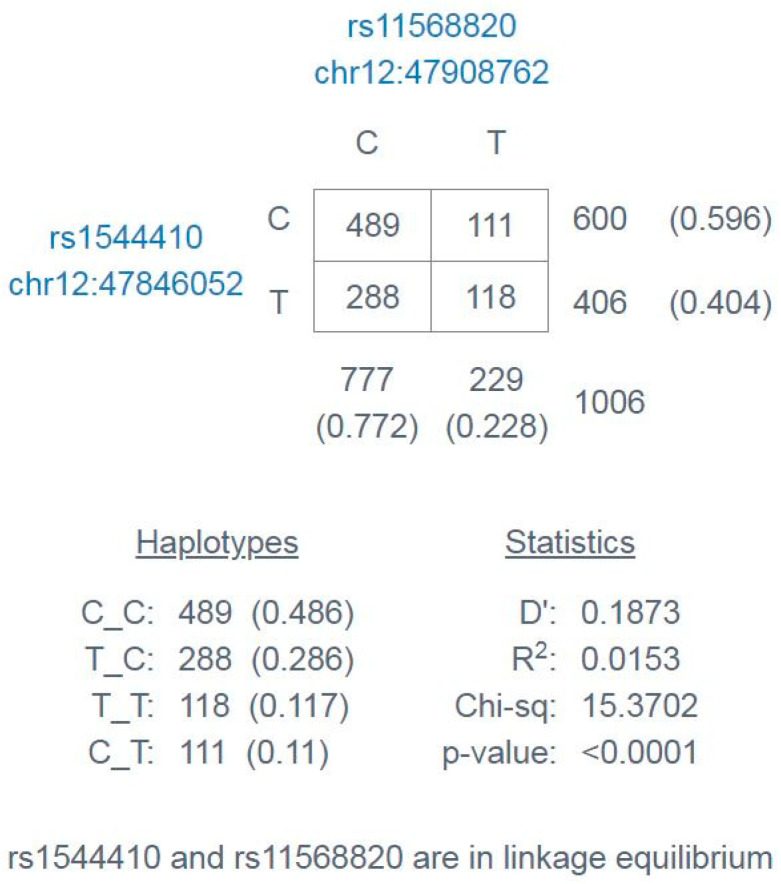
Linkage disequilibrium analysis and haplotype frequencies for European populations from the 1000Genomes Project database.

Using the LD Link program, linkage disequilibrium analysis of these loci was also performed for five European populations (CEU, GBR, FIN, IBS, TSI) from the “1000Genomes Project” database, and the result was presented in Figure 2. For the European populations, linkage disequilibrium was also obtained for rs1544410 and rs11568820 of the *VDR* gene.

Analyzing the frequency of co-occurrence of genotypes of *VDR* gene polymorphisms in groups distinguished based on T-score values, it was observed that the co-occurrence of genotypes GG (rs1544410) with GG (rs11568820) statistically significantly increases the risk of osteopenia 35.7% vs. 23.1% in the control group; OR = 1.85 95%CI: 1.051–3.244; *p* = 0.032. In the group with osteoporosis, such a combination of genotypes occurred in 32.0% of the examined women and was also higher than that observed in women with normal bone density, but without statistical significance (OR = 1.56; 95%CI: 0.961–2.542; *p* = 0.071). The total occurrence of the remaining genotypes of the studied *VDR* gene variants did not show statistically significant differences between the analyzed groups of women (Table 4).

## 3. Discussion

Maintaining mineral homeostasis in the human body requires the interaction of several factors, such as parathyroid hormone and vitamin D. Moreover, one of the key components of the endocrine system is *VDR* which regulates numerous biological processes. *VDR* is not only involved in calcium homeostasis, but also differentiation, and immune system modulation [14]. *VDR* belongs to the nuclear receptor and acts as a ligand-activated transcription factor. In response to hormone binding, *VDR* heterodimerizes with the retinoic X receptor (RXR), resulting in a change in its spatial conformation. Then, the heterodimer binds to the respective promoter sites of vitamin D-dependent genes and regulators gene transcription, by their activation or suppression [15].

Molecular studies of the *VDR* gene showed that mutations in this gene are associated with type II vitamin D-resistant rickets [16]. To date, almost 1.000 polymorphic DNA sequence variants in the *VDR* locus have been reported. There are many studies showing linkage of these polymorphisms to various diseases. The *VDR* genetic variants have been associated with a predisposition to metabolic bone diseases, chronic diseases, cancer, autoimmune diseases, cardiovascular alterations, rheumatic arthritis, and diabetes [17]. The best studied *VDR* gene polymorphisms are *Apal* (rs7975232, A>C), *BsmI* (rs1544410, A>G), *Taql* (rs731236, C>T), *Fokl* (rs10735810, C>T), and *Cdx2* (rs11568820, G>A). Those SNPs are located near the 3′ end of the gene and increase mRNA stability. It was shown that the *BsmI* variant is associated with a different length of a poly(A) tail [11]. The *FokI* polymorphism is located in the 5′ coding region of the exon 2 and creates a new start codon resulting in a protein shortened by three amino acids. This shorter receptor presents higher transcriptional activity than the longer isoform [18]. The *Cdx2* polymorphism seriously influences the transcriptional activity of the vitamin D receptor that binds the responsive elements of target genes. It was also shown that individuals with the AA genotype of the *Cdx2* polymorphism also had lower levels of *VDR* gene expression [10].

So far, some studies conducted on the Polish population regarding *VDR* polymorphisms have been established. Dębniak et al. identified an association between *Fokl* SNP and increased early-onset breast cancer risk [19]. It was found that *BsmI* in the dominant inheritance model has a moderate risk of the development of ovarian cancer [20]. Moreover, it was shown that *BsmI* polymorphism is associated with some parameters of carbohydrate metabolism in Polish people aged over 65 [21]. The *VDR* polymorphisms were also connected to Graves’ disease in the Polish population. It was demonstrated that the “bb” genotype of *BsmI* polymorphism and the “FF” genotype of FokI polymorphism increased the risk of development of this autoimmune thyroid disorder [22]. It is well known that vitamin D has a significant effect on the modulation of inflammatory mechanisms. Krela-Kaźmierczak et al. studied the relationship between two *VDR* variants (*ApaI* and *FokI*), serum vitamin D concentration, and bone mineral density in Polish patients with inflammatory bowel disease (IBD). The statistically significant differences in bone mineral density in the lumbar spine and the closer end of the femoral neck were observed among carriers of the same polymorphic variants but presented different IBD types (Crohn’s disease, ulcerative colitis) [23].

A few reports showed the connection of *VDR* SNPs to osteoporosis in the Polish population. The study conducted on a large cohort of postmenopausal Polish women from the Silesia region indicated that the AG heterozygous genotype of *TaqI* determines lower bone mineral density values [24]. There was no difference in age, vitamin D3 concentration, waist circumference, or BMI between carriers of a particular genotype [24]. In contrast, a study based on postmenopausal Polish women from the Great Poland region indicated that patients with heterozygous genotype of *BsmI* polymorphism presented higher bone densitometry parameters, both before and after monoclonal antibody against cytokine RANKL treatment (denosumab) [25].

The association of *VDR* gene polymorphisms with osteoporosis is complex and inconclusive. To clarify the contribution of *VDR* polymorphisms to genetic susceptibility to osteoporosis and osteopenia among Polish patients, especially from the West Pomeranian population, we conducted a case-control study by analyzing two well-characterized *VDR* polymorphisms. We reported no evidence of the association between the *BsmI* (rs1544410, A>G) and *Cdx2* (rs11568820, G>A) polymorphisms of the *VDR* gene with those two conditions that affect bone density and increased fracture risk in the Polish population of women with osteoporosis and osteopenia. For both polymorphisms rs11568820 and rs11568820, the less common A allele occurred more frequently in the control group without statistical significance. Moreover, logistic regression in different inheritance models showed that genotypes containing the A allele of the rs1544410 variant were more frequent in the control group than among patients with osteopenia. In the second variant rs11568820 of the *VDR* gene, the GG genotype occurred more frequently in women with osteopenia than in the control group. No statistically significant differences were shown in genotypes containing at least one mutated A allele between women with osteopenia and controls, although they were more frequent in the control group than in the osteopenia group. As in the case of osteopenia, no statistically significant differences were observed between genotypes of *VDR* gene variants and the risk of osteoporosis. However, as in the case of osteopenia, a higher incidence of genotypes containing the mutated A allele was observed in the control group.

Many previous studies have investigated the association between *VDR* polymorphisms and osteoporosis risk. Many of them demonstrated contradictory results. Some results confirmed the association of *VDR* polymorphisms with the risk of osteoporosis, others showed no association with this condition. For example, Jia et al. examined 4594 cases with osteoporosis and 5527 controls to show that the *VDR BsmI* polymorphism was associated with a decreased osteoporosis risk [26]. However, Gang et al. indicated that the *VDR BsmI* polymorphism was not significantly associated with osteoporosis risk [27]. Moreover, some reports suggest the association of *VDR* variants with osteoporosis in a particular population [28]. Our results are in agreement with a meta-analysis that presented a lack of significant association between the *VDR* polymorphisms *BsmI* and *Cdx2* with osteoporotic fracture risk [28,29,30].

Nonetheless, the linkage disequilibrium (LD) analysis for studied loci based on five European populations (CEU, GBR, FIN, IBS, TSI) pointed out that the co-appearance of both genotypes GG (rs1544410) and GG (rs11568820) statistically significantly increased the prevalence of osteopenia. In the group with osteoporosis, on the other hand, co-appearance was not statistically significant.

An interesting result was obtained in the association analysis of *VDR* alleles and genotypes with densitometric results for women with osteoporosis. The comparing median Z-scores in patients with osteoporosis for the rs11568820 variant gave a median of −2.19 (−2.33; −1.58) for the G allele and −1.62 (−2.04; −1.17) for the A allele, with statistically significant difference *p* = 0.027. In addition, for the rs11568820 SNP, medians of the T-score and Z-score were lower among patients with osteoporosis with GG than AA genotypes but were not statistically significant. These results are in line with earlier studies. Marozik et al. showed that the *Cdx2* AA genotype is associated with increased bone mineral density in a Belarusian population of postmenopausal women [31]. Similar results were obtained among postmenopausal Japanese women. Arai et al. revealed that the bone mineral density in the lumbar spine (L2-L4) with the Cdx-G homozygote was 12% lower than with the Cdx-A homozygote (*p* < 0.05) [32]. In contrast to the above-mentioned results, Gentil et al. demonstrated that in postmenopausal women, the *Cdx2* polymorphism is not the only factor affecting bone mineral density, but it is strongly correlated with physical activity. Active women with the *Cdx2* GG genotype showed higher adjusted BMD of the femoral neck and Ward’s triangle than inactive women with the same genotype [33]. Morita et al. also noted a significant interaction between Cdx2 genotypes and sports activity. A significantly higher lumbar spine BMD was detected among physically active postmenopausal women with the Cdx GG genotype, than in sport-inactive women [34].

In addition, a statistically significant association of the age of the last period with the AA genotype was found in the osteopenic and control groups for rs11568820 polymorphism. In the osteopenia group, the mean age at last menstruation was 49.1 ± 4.1 in patients with the GG genotype, 50.4 ± 3.9 in GA, and 44.0 ± 5.3 in AA (*p* = 0.057), while in the control group it was 50.8 ± 4.2 in patients with the GG genotype, 50.2 ± 3.7 in heterozygotes, and 43.3 ± 2.1 in women with the AA genotype (*p* = 0.014). Among patients with osteoporosis, a similar correlation was noted, but there was no statistical importance. Till now, the above correlation between lower age at the onset of the last menstrual period and AA rs11568820 has not yet been described in the literature on osteoporosis. A study on this correlation requires further analysis. It should be highlighted that *VDR* polymorphisms themself are not the only determinant of osteoporosis risk. Many other factors such as age, gender, diet, lifestyle, and other genetic predispositions influence the development of the disease [35].

## 4. Materials and Methods

### 4.1. Study Group

Patients were recruited for the study during bone mineral density tests at the Densitometry Laboratory, Clinical Hospital No. 1 of the Pomeranian Medical University in Szczecin. Written consent was obtained from 442 women. Based on the T-score results, they were classified into three groups: 147 (33.26%) with normal bone density (control group), 98 (22.17%) with T-score from −1 to −2.5 (osteopenia), and 197 (44.57%) with T-score below −2.5 (osteoporosis). Each patient was interviewed to obtain the necessary information on the presence of the disease, use of medications, age at first and last menstruation, number of pregnancies, and birth weight. The study included women who had reached menopause at least one year earlier and had not undergone therapy affecting bone mass (selective estrogen receptor modulators—SERM, calcitonin, biphosphates, heparin, steroids, thyroid hormones, antiepileptic drugs, GnRH analogs, tibolone, or hormone replacement therapy (HRT)). Patients after bilateral ovariectomy, as well as women suffering from endocrine and metabolic disorders, hematological diseases, neoplastic diseases, kidney diseases, autoimmune diseases, or connective tissue diseases, were excluded from the study due to the possibility that these diseases affect bone loss.

A comparison of the results obtained in the densitometric examination for the individual groups of women is presented in Table 5. The median for bone mineral density (BMD) was 1.18 g/cm^3^ in the control group, 0.97 g/cm^3^ in osteopenia and 0.83 g/cm^3^ in osteoporosis (*p* < 0.001). The median BMD as % of peak bone mass (BMD%YA) and as % of age norm (BMD%AM) also statistically significantly differed between the groups (for controls: 98% and 107%, for osteopenia: 81% and 88%, and for osteoporosis: 69% and 78%, respectively). The following values for T-score were obtained: control group −0.17, osteopenia −1.91, and osteoporosis −3.05 (*p* < 0.001). Z-scores were 0.56, −0.94, and −1.62 for control, osteopenia, and osteoporosis, respectively (*p* < 0.001) (Table 5).

Table 6 summarizes the characteristics of the clinical data of the patients from the studied groups. The mean age in the control group was 53.58 ± 8.26 years. In the case of osteopenia, it was 52.92 ± 8.04 years, and in the case of osteoporosis, it was 56.64 ± 8.81 years (*p* = 0.003). Patients with osteoporosis had on average a statistically significantly lower body weight: 61.04 ± 9.18 kg vs. 65.25 ± 11.18 kg in osteopenia (*p* = 0.027) and 68.53 ± 12.31 kg in osteoporosis (*p* = 0.0001).

When calculating the body mass index, the highest mean was obtained for the control group 25.88 ± 4.65 kg/m^2^, and the lowest for patients with osteoporosis 23.72 ± 3.11 kg/m^2^ (*p* = 0.003). Classifying the obtained BMI values into four categories: underweight, normal weight, overweight, and obesity, it was observed that patients with osteoporosis were most often underweight (4.57% vs. 2.04% in osteopenia and 1.36% in control), and least often obese (2.10% vs. 5.10% in osteopenia and 17.69% in control) (Table 6).

No statistically significant differences were observed when comparing the average number of pregnancies and the time of the first menstrual period. However, the last menstrual period occurred on average earlier in women with osteoporosis at 48.05 ± 5.08 years compared to the control group at 50.38 ± 4.24 years (*p* = 0.028). Statistically significant differences were also found between the years since menopause, the average of which in osteoporosis was 10.63 ± 5.75 years, and for osteopenia 7.09 ± 6.19 (*p* = 0.004) and 7.03 ± 5.59 in the control group (0.014). When analyzing the newborn weights, the lowest averages were obtained in the groups with osteoporosis 3141.25 ± 536.32 g and osteopenia 3226.79 ± 411.07 g. Both groups were statistically significantly different from the average obtained for the control group 3628.95 ± 480.75 g.

### 4.2. DNA Isolation and Genotyping

Genomic DNA was isolated from venous blood using a QIAamp DNA Blood Mini Kit (Qiagen, Hilden, Germany) according to the manufacturer’s protocol. Genotypes for rs1544410 and rs11568820 were determined by real-time PCR using the SimpleProbe^®^. Primers and probes were designed by TIB Molbiol (Berlin, Germany). PCR was performed in a 20 μL reaction volume containing 50 ng of genomic DNA, 4 μL of 5× LightCycler^®^ 480 Genotyping Master (Roche Diagnostics, Mannheim, Germany), 1ul of primers, and Fluorescein-conjugated probe and 2.5 mmol/L MgCl_2_. PCR thermal cycling conditions were as follows: denaturation for 10 min at 95 °C followed by 45 cycles, including denaturation at 95 °C for 10 s; primer annealing at 60 °C for 10 s and elongation at 70 °C for 15 s. The fluorescence reading was taken at the end of each 72 °C step. A melting curve was generated at the end of the amplification cycles by increasing the temperature from 40 °C to 75 °C at 0.2 °C/s. The PCR amplification was conducted using a LightCycler 96 instrument (Roche Diagnostics, Mannheim, Germany). Next, the LightCycler^®^ 96 Software (Version 1.2) Genotyping module was grouped into wild-type, heterozygotes, and mutants based on a melting curve.

### 4.3. Statistical Analysis

Statistical analysis was performed using the R program (version 4.2.2) [36] and the “SNPassoc” package (version 2.0-2) [37]. In the first stage, the normality of data distributions on an interval scale was checked using the Shapiro–Wilk test. Parametric variables were presented as arithmetic means with standard deviation (±SD), and nonparametric variables were presented as medians with lower and upper quartiles (Q1; Q3). Analysis of variance (ANOVA) and Tukey’s post-hoc test were used to compare parametric variables. The Kruskal–Wallis test was used to compare nonparametric data, and pairwise comparisons were performed using the Wilcoxon rank-sum test with continuity correction. The χ2 test or Fisher’s exact test was used to analyze nominal variables, and they were presented as numbers and percentages. To assess the correlation between clinical and densitometric data, Spearman’s rho correlation coefficient was used for nonparametric data with Holm’s correction. Unconditional logistic regression was used to calculate odds ratios (OR) with 95% confidence intervals to assess the strength of association between *VDR* gene polymorphisms and the risk of osteopenia and osteoporosis. Odds ratios were calculated for the comparison of allele frequencies (G vs. A) and genotypes in five genetic models: codominant (AA vs. GA vs. GG), dominant (AA + GA vs. GG), recessive (GG + GA vs. AA), overdominant (AA + GG vs. GA), and log-additive (1,2,3). The models were assessed using the Akaike information criterion (AIC), and statistical significance was set at *p* < 0.05. Haplotype analysis was performed using HaploView 4.2 software.

## 5. Conclusions

The obtained results complement studies on the association of *VDR* gene polymorphisms with the risk of osteopenia and osteoporosis in Polish women. These studies require further large-scale studies. To date, the results suggest that *VDR* genetic variants may influence the risk of developing osteoporosis, but that this influence may vary according to population, gender, and other factors. However, in our study, no association was found between the examined *VDR* polymorphisms (rs1544410, rs11568820) and the risk of osteoporosis.

## Figures and Tables

**Table 1:** Comparative analysis of densitometric data depending on genotypes in the study groups.

Parameter	Median (Q1; Q3)	Median (Q1; Q3)	Median (Q1; Q3)	*p*
Control N = 147				
rs1544410	GG	GA	AA	*p*
L2L4 BMD	1.17 (1.10; 1.20)	1.22 (1.16; 1.27)	1.15 (1.09; 1.22)	0.249
L2L4 YA	98.00 (93.00; 100.00)	101.00 (96.00; 106.00)	95.50 (91.00; 101.00)	0.258
L2L4 AM	108.00 (102.00; 113.00)	110.00 (103.00; 119.00)	105.50 (93.00; 106.00)	0.434
T-score	−0.22 (−0.79; 0.00)	0.14 (−0.35; 0.57)	−0.45 (−0.89; 0.15)	0.219
Z-score	0.71 (−0.11; 1.23)	0.58 (0.39; 1.43)	0.10 (−0.53; 0.38)	0.468
rs11568820	GG	GA	AA	*p*
L2L4 BMD	1.13 (1.13; 1.13)	1.19 (1.12; 1.25)	1.19 (1.12; 1.25)	0.702
L2L4 YA	94.00 (94.00; 94.00)	99.50 (95.00; 104.50)	99.00 (94.00; 104.00)	0.677
L2L4 AM	93.00 (93.00; 93.00)	107.50 (99.50; 113.50)	107.50 (103.00; 115.00)	0.318
T-score	−0.62 (−0.62; −0.62)	−0.21 (−0.67; 0.27)	−0.17 (−0.66; 0.52)	0.729
Z-score	−0.68 (−0.68; −0.68)	0.76 (−0.20; 1.43)	0.58 (0.10; 1.19)	0.327
Osteopenia N = 98				
rs1544410	GG	GA	AA	*p*
L2L4 BMD	0.95 (0.92; 1.02)	0.99 (0.93; 1.04)	0.95 (0.93; 0.98)	0.361
L2L4 YA	79.00 (77.00; 85.00)	82.50 (77.50; 87.00)	79.50 (77.50; 81.50)	0.330
L2L4 AM	90.00 (84.00; 94.00)	90.00 (84.00; 97.50)	90.50 (86.50; 92.50)	0.784
T-score	−2.06 (−2.30; −1.52)	−1.75 (−2.25; −1.32)	−2.06 (−2.22; −1.87)	0.371
Z-score	−0.88 (−1.44; −0.52)	−0.92 (−1.38; −0.26)	−0.94 (−1.18; −0.83)	0.887
rs11568820	GG	GA	AA	*p*
L2L4 BMD	0.93 (0.92; 0.97)	1.01 (0.94; 1.04)	0.96 (0.93; 1.02)	0.420
L2L4 YA	78.00 (77.00; 81.00)	84.00 (78.50; 87.00)	80.00 (77.00; 85.50)	0.365
L2L4 AM	82.00 (81.00; 89.50)	91.00 (85.00; 94.50)	87.00 (84.00; 93.00)	0.293
T-score	−2.22 (−2.29; −1.93)	−1.61 (−2.13; −1.35)	−1.93 (−2.20; −1.42)	0.486
Z-score	−1.74 (−1.85; −1.00)	−0.76 (−1.50; −0.02)	−0.98 (−1.31; −0.68)	0.430
Osteoporosis N = 197				
rs1544410	GG	GA	AA	*p*
L2L4 BMD	0.83 (0.75; 0.86)	0.82 (0.77; 0.88)	0.82 (0.79; 0.88)	0.873
L2L4 YA	70.00 (62.00; 71.00)	68.00 (64.00; 73.00)	68.00 (66.50; 73.50)	0.729
L2L4 AM	79.00 (76.00; 82.00)	80.00 (75.00; 84.00)	76.00 (74.50; 81.50)	0.805
T-score	−3.04 (−3.76; −2.87)	−3.15 (−3.55; −2.71)	−3.16 (−3.39; −2.63)	0.915
Z-score	−1.78 (−2.04; −1.15)	−1.53 (−2.00; −1.29)	−1.91 (−2.33; −1.46)	0.559
rs11568820	GG	GA	AA	*p*
L2L4 BMD	0.75 (0.74; 0.76)	0.83 (0.73; 0.87)	0.83 (0.79; 0.87)	0.294
L2L4 YA	63.00 (62.00; 64.00)	69.00 (63.50; 72.50)	69.00 (66.00; 73.00)	0.293
L2L4 AM	74.00 (73.00; 75.00)	78.00 (75.00; 79.50)	79.00 (74.00; 83.00)	0.505
T-score	−3.71 (−3.80; −3.63)	−3.10 (−3.94; −2.72)	−3.05 (−3.40; −2.71)	0.232
Z-score	−2.47 (−2.68; −2.26)	−1.91 (−2.19; −1.53)	−1.62 (−2.03; −1.15)	0.120

*p* Kruskal–Wallis rank sum test.

**Table 2:** Comparative analysis of clinical data depending on genotypes in the study groups.

Parameter	Mean ± SD	Mean ± SD	Mean ± SD	*p*
Control N = 147				
rs1544410	GG	GA	AA	*p*
Age (years)	57.4 ± 5.9	56.8 ± 5.9	53.2 ± 7.1	0.365
BMI (kg/m^2^)	26.1 ± 3.9	28.5 ± 4.9	26.3 ± 5.8	0.387
Newborn weight (g)	3552.9 ± 152.7	3698.9 ± 685.4	3596.7 ± 295.0	0.843
Menarche (years)	13.1 ± 2.1	13.6 ± 1.9	13.4 ± 0.9	0.818
Last menstrual period (years)	50.7 ± 3.2	48.8 ± 4.7	48.4 ± 5.0	0.407
Years since menopause	38.1 ± 5.4	35.1 ± 5.4	35.0 ± 4.6	0.295
***rs11568820***	***GG***	***GA***	***AA***	*p*
Age (years)	54.3 ± 8.6	52.0 ± 7.4	50.0 ± 7.8	0.579
BMI (kg/m^2^)	26.2 ± 5.0	24.9 ± 3.6	29.7 ± 4.2	0.448
Newborn weight (g)	3741.7 ± 474.5	3360.0 ± 453.7	3890.0 ± 1387	0.254
Menarche (years)	13.7 ± 1.8	12.7 ± 2.0	12.7 ± 0.6	0.297
Last menstrual period (years)	50.8 ± 4.2	50.2 ± 3.7	43.3 ± 2.1	0.014
Years since menopause	35.8 ± 5.1	38.4 ± 5.4	28.0 ± 5.2	0.127
Osteopenia N = 98				
rs1544410	GG	GA	AA	*p*
Age (years)	55.1 ± 4.9	56.4 ± 7.1	57.0 ± 8.1	0.683
BMI (kg/m^2^)	24.8 ± 2.1	25.9 ± 5.6	23.9 ± 3.6	0.409
Newborn weight (g)	3217.9 ± 491.6	3215.5 ± 355.4	3310.0 ± 259.4	0.938
Menarche (years)	13.3 ± 2.7	12.8 ± 2.1	12.2 ± 2.0	0.526
Last menstrual period (years)	49.6 ± 3.3	48.3 ± 5.7	49.6 ± 3.2	0.593
Years since menopause	35.8 ± 4.2	35.8 ± 6.3	37.4 ± 3.0	0.719
rs11568820	GG	GA	AA	*p*
Age (years)	52.7 ± 8.2	54.0 ± 7.9	51.7 ± 7.6	0.770
BMI (kg/m^2^)	24.6 ± 3.9	25.1 ± 4.3	23.8 ± 3.3	0.818
Newborn weight (g)	3199.0 ± 317.7	3396.7 ± 667.7	2995.0 ± 346.5	0.432
Menarche (years)	12.9 ± 2.5	13.1 ± 1.7	12.7 ± 2.9	0.949
Last menstrual period (years)	49.1 ± 4.1	50.4 ± 3.9	44.0 ± 5.3	0.057
Years since menopause	35.9 ± 4.9	37.6 ± 4.6	31.3 ± 2.5	0.132
Osteoporosis N = 197				
rs1544410	GG	GA	AA	*p*
Age (years)	59.3 ± 6.1	60.1 ± 5.4	57.7 ± 6.3	0.425
BMI (kg/m^2^)	23.8 ± 2.9	23.9 ± 2.9	24.1 ± 3.3	0.936
Newborn weight (g)	3103.3 ± 711.2	3056.7 ± 366.9	3210.0 ± 492.1	0.909
Menarche (years)	12.3 ± 1.9	13.3 ± 2.2	13.1 ± 2.4	0.295
Last menstrual period (years)	48.1 ± 4.4	48.2 ± 5.4	48.9 ± 4.9	0.887
Years since menopause	36.1 ± 4.2	35.2 ± 5.3	35.7 ± 5.7	0.797
rs11568820	GG	GA	AA	*p*
Age (years)	56.5 ± 9.0	57.1 ± 8.5	58.5 ± 6.4	0.916
BMI (kg/m^2^)	23.8 ± 3.2	23.6 ± 2.4	24.1 ± 3.8	0.975
Newborn weight (g)	3160.8 ± 578.7	3250.0 ± 212.1	3500 ± 248.1	0.835
Menarche (years)	12.9 ± 2.2	12.9 ± 1.9	13.5 ± 2.1	0.928
Last menstrual period (years)	47.9 ± 5.2	48.9 ± 4.5	46.0 ± 8.5	0.716
Years since menopause	35.6 ± 5.0	36.3 ± 4.4	32.5 ± 10.6	0.631

*p* Anova.

**Table 3:** Estimated frequency of haplotypes in the studied groups.

Haplotype rs1544410/rs11568820	Frequency				*p* Control vs. Osteopenia	*p* Control vs. Osteoporosis
	All Group	Osteopenia	Osteoporosis	Control		
GG	0.537	0.570	0.554	0.491	0.100	0.129
AG	0.319	0.292	0.314	0.345	0.244	0.452
GA	0.087	0.088	0.078	0.100	0.728	0.399
AA	0.057	0.049	0.054	0.063	0.455	0.473

*p* chi square.

**Table 4:** Frequencies of co-occurrence of genotypes of rs1544410 and rs11568820 polymorphisms of the *VDR* gene in the studied groups.

Control	Genotypes, n (%)				
	rs1544410	rs11568820			
		GG	GA	AA	Total
	GG	34 (23.1)	16 (10.9)	2 (1.4)	52 (35.4)
	GA	52 (35.4)	18 (12.2)	0 (0.0)	70 (47.6)
	AA	17 (11.6)	6 (4.1)	2 (1.4)	25 (17.0)
	Total	103 (70.1)	40 (27.2)	4 (2.7)	147 (100.0)
Osteopenia	rs1544410	rs11568820			
		GG	GA	AA	Total
	GG	35 (35.7)	10 (10.2)	1 (1.0)	46 (46.9)
	GA	27 (27.6)	9 (9.2)	1 (1.0)	37 (37.8)
	AA	12 (12.2)	2 (2.0)	1 (1.0)	15 (15.3)
	Total	74 (75.5)	21 (21.4)	3 (3.1)	98 (100.0)
Osteoporosis	rs1544410	rs11568820			
		GG	GA	AA	Total
	GG	63 (32.0)	20 (10.2)	0 (0.0)	83 (42.1)
	GA	63 (32.0)	17 (8.6)	3 (1.5)	83 (42.1)
	AA	23 (11.7)	7 (3.6)	1 (0.5)	31 (15.7)
	Total	149 (75.6)	44 (22.3)	4 (2.0)	197 (100.0)

**Table 5:** Comparison of the results obtained in the DXA study in patients.

Parameter	Control N = 147	Osteopenia N = 98	Osteoporosis N = 197	*p* ANOVA Kruskala-Wallisa	*p* Control vs. Osteopenia	*p* Control vs. Osteoporosis	*p* Osteopenia vs. Osteoporosis
L2L4 BMD (g/cm^2^)	1.18 (1.12; 1.25) 1.21 ± 0.10	0.97 (0.93; 1.03) 0.98 ± 0.05	0.83 (0.77; 0.87) 0.82 ± 0.07	<0.001	<0.001	<0.001	<0.001
L2L4 YA (%)	98.00 (94.00; 104.00) 100.47 ± 8.22	81.00 (77.00; 86.00) 81.41 ± 4.41	69.00 (65.00; 73.00) 68.09 ± 5.40	<0.001	<0.001	<0.001	<0.001
L2L4AM (%)	107.00 (101.50; 114.00) 108.73 ± 10.39	88.00 (84.00; 94.00) 88.76 ± 6.43	78.00 (74.00; 82.50) 78.08 ± 7.16	<0.001	<0.001	<0.001	<0.001
T-score	−0.17 (−0.67; 0.45) 0.05 ± 0.91	−1.91 (−2.21; −1.42) −1.83 ± 0.44	−3.05 (−3.47; −2.71) −3.18 ± 0.55	<0.001	<0.001	<0.001	<0.001
Z-score	0.56 (−0.10; 1.33) 0.64 ± 1.11	−0.94 (−1.44; −0.52) −0.94 ± 0.61	−1.62 (−2.12; −1.18) −3.57 ± 15.69	<0.001	<0.001	<0.001	<0.001

The results are presented as median (lower and upper quartile) and arithmetic mean values ± standard deviation.

**Table 6:** Characteristics of the study population.

Parameter	Control N = 147	Osteopenia N = 98	Osteoporosis N = 197	*p*	*p* Control vs. Osteopenia	*p* Control vs. Osteoporosis	*p* Osteopenia vs. Osteoporosis
Age (years), mean ± SD Age, n (%) <40 40–49 50–59 60–69 ≥70	53.58 ± 8.26 7 (4.76) 31 (21.09) 76 (51.70) 28 (19.05) 5 (3.40)	52.92 ± 8.04 5 (5.10) 21 (21.43) 58 (59.18) 10 (10.21) 4 (4.08)	56.64 ± 8.81 5 (2.54) 26 (13.20) 84 (42.64) 74 (37.56) 8 (4.06)	0.003 χ^2^ = 32.36 <0.001	0.880 χ^2^ = 3.66 0.454	0.052 χ^2^ = 15.67 0.003	0.003 χ^2^ = 24.98 <0.001
Body mass (kg), mean ± SD	68.53 ± 12.31	65.25 ± 11.18	61.04 ± 9.18	<0.001	0.153	0.0001	0.027
Height (cm), mean ± SD	162.87 ± 5.72	162.74 ± 5.19	160.25 ± 5.16	0.002	0.987	0.010	0.005
BMI (kg/m^2^), mean ± SD BMI, n (%) <18.5 underweight 18.5–24.99 normal weight 25–29.99 overweight ≥30 obesity	25.88 ± 4.65 2 (1.36) 76 (51.70) 43 (29.25) 26 (17.69)	24.64 ± 3.95 2 (2.04) 47 (47.96) 44 (44.90) 5 (5.10)	23.72 ± 3.11 9 (4.57) 120 (60.91) 63 (31.98) 5 (2.54)	0.005 χ^2^ = 35.51 <0.001	0.125 χ^2^ = 11.74 0.008	0.003 χ^2^ = 25.605 <0.001	0.254 χ^2^ = 7.3417 0.062
Menarche (years), mean ± SD	13.38 ± 1.88	12.91 ± 2.35	12.94 ± 2.16	0.582	0.602	0.623	0.997
Last menstrual period (years), mean ± SD	50.38 ± 4.24	49.14 ± 4.21	48.05 ± 5.08	0.034	0.381	0.028	0.344
Years since menopause, mean ± SD	7.03 ± 5.59	7.09 ± 6.19	10.63 ± 5.75	0.002	0.999	0.014	0.004
Number of pregnancies, mean ± SD median (Q1; Q3)	1.98 ± 1.22 2 (1–3) *	1.79 ± 1.08 2 (1–3) *	1.93 ± 1.32 2 (1–3) *	0.758 *	0.800	0.800	0.800
Newborn weight (g), mean ± SD	3628.95 ± 480.75	3226.79 ± 411.07	3141.25 ± 536.32	0.005	0.014	0.009	0.828

*p*-Anova, * *p*-Kruskal–Wallis rank sum test.

## Data Availability

The original contributions presented in this study are included in the article. Further inquiries can be directed to the corresponding author.

## Supplementary Materials

The following supporting information can be downloaded at: https://www.mdpi.com/article/10.3390/ijms26020481/s1.
