# Supplementary material for: The VDR rs1544410 and rs11568820 Variants and the Risk of Osteoporosis in the Polish Population
Source: Int J Mol Sci. 2025 Jan 8;26(2):481. doi: 10.3390/ijms26020481 (PMC11764738; doi:10.3390/ijms26020481)
Supplement: Supplementary file 1 [file ijms-26-00481-s001.zip › ijms-3355193-supplementary.pdf]

International Journal of
Molecular Sciences

MDPI

Supplementary Materials

The VDR rs1544410 and rs11568820 Variants and the Risk of Osteoporosis in the Polish Population

Adam Kamiński [1], Anna Bogacz [2,3,*], Joanna Niezgoda [4], Marta Podralska [3], Aleksandra Górska [3], Michał Soczawa [5] and Bogusław Czerny [3,4]

1 Department of Orthopaedics and Traumatology, Independent Public Clinical Hospital No. 1, Pomeranian Medical University in Szczecin, Unii Lubelskiej 1, 71-252 Szczecin, Poland; emluc@wp.pl (A.K.)
2 Department of Physiology, Poznan University of Medical Sciences, Święcickiego 6, 60-781 Poznan, Poland
3 Department of Stem Cells and Regenerative Medicine, Institute of Natural Fibres and Medicinal Plants, Kolejowa 2, 62-064 Plewiska, Poland; marta.podralska@iwnirz.pl (M.P.); aleksandra.gorska@iwnirz.pl (A.G.)
4 Department of Pharmacology and Pharmacoeconomics, Pomeranian Medical University in Szczecin, 71-230 Szczecin, Poland; lek.joanna.niezgoda@gmail.com
5 Department and Clinic of Urology and Urological Oncology, Pomeranian Medical University in Szczecin, al. Powstańców Wielkopolskich 72, 70-111 Szczecin, Poland; michal.soczawa@pum.edu.pl (M.S.)
* Correspondence: aniabogacz23@o2.pl

Table S1. Association between the risk of osteopenia and the *VDR* rs1544410 and rs11568820 polymorphisms.

SNP	Model	genotype	control	osteopenia	OR (95%CI)	*p*	AIC
rs1544410	codominant	***GG***	52 (35.4)	46 (46.9)	1.00	0.185	332.4
		GA	70 (47.6)	37 (37.8)	0.60 (0.34-1.05)		
		AA	25 (17.0)	15 (15.3)	0.68 (0.32-1.44)		
	dominant	***GG***	52 (35.4)	46 (46.9)	1.00	0.071	330.5
		GA-AA	95 (64.6)	52 (53.1)	0.62 (0.37-1.04)		
	recessive	***GG-GA***	122 (83.0)	83 (84.7)	1.00	0.723	333.7
		AA	25 (17.0)	15 (15.3)	0.88 (0.44-1.77)		
	overdominant	***GG-AA***	77 (52.4)	61 (62.2)	1.00	0.126	331.4
		GA	70 (47.6)	37 (37.8)	0.67 (0.40-1.12)		
	log-additive	***0,1,2***	147 (60.0)	98 (40.0)	0.77 (0.53-1.10)	0.152	331.7
rs11568820	codominant	***GG***	103 (70.1)	74 (75.5)	1.00	0.586	334.7
		GA	40 (27.2)	21 (21.4)	0.73 (0.40-1.34)		
		AA	4 (2.7)	3 (3.1)	1.04 (0.23-4.80)		
	dominant	***GG***	103 (70.1)	74 (75.5)	1.00	0.349	332.9
		GA-AA	44 (29.9)	24 (24.5)	0.76 (0.43-1.36)		
	recessive	***GG-GA***	143 (97.3)	95 (96.9)	1.00	0.876	333.8
		AA	4 (2.7)	3 (3.1)	1.13 (0.25-5.16)		
	overdominant	***GG-AA***	107 (72.8)	77 (78.6)	1.00	0.302	332.7
		GA	40 (27.2)	21 (21.4)	0.73 (0.40-1.33)		
	log-additive	***0,1,2***	147 (60.0)	98 (40.0)	0.82 (0.50-1.36)	0.449	333.2

Table S2. Association between the risk of osteoporosis and the *VDR* rs1544410 and rs11568820 polymorphisms.

SNP	Model	Genotype	Control	Osteoporosis	OR (95%CI)	*p*	AIC
rs1544410	codominant	***GG***	52 (35.4)	83 (42.1)	1.00	0.441	474.0
		GA	70 (47.6)	83 (42.1)	0.74 (0.46-1.19)		

		AA	25 (17.0)	31 (15.7)	0.78 (0.41-1.46)		
	dominant	***GG***	52 (35.4)	83 (42.1)	1.00	0.203	472.0
		GA-AA	95 (64.6)	114 (57.9)	0.75 (0.48-1.17)		
	recessive	***GG-GA***	122 (83.0)	166 (84.3)	1.00	0.753	473.5
		AA	25 (17.0)	31 (15.7)	0.91 (0.51-1.62)		
	overdominant	***GG-AA***	77 (52.4)	114 (57.9)	1.00	0.311	472.6
		GA	70 (47.6)	83 (42.1)	0.80 (0.52-1.23)		
	log-additive	***0,1,2***	147 (42.7)	197 (57.3)	0.85 (0.63-1.15)	0.299	472.5
rs11568820	codominant	***GG***	103 (70.1)	149 (75.6)	1.00	0.511	474.3
		GA	40 (27.2)	44 (22.3)	0.76 (0.46-1.25)		
		AA	4 (2.7)	4 (2.0)	0.69 (0.17-2.83)		
	dominant	***GG***	103 (70.1)	149 (75.6)	1.00	0.250	472.3
		GA-AA	44 (29.9)	48 (24.4)	0.75 (0.47-1.22)		
	recessive	***GG-GA***	143 (97.3)	193 (98.0)	1.00	0.676	473.4
		AA	4 (2.7)	4 (2.0)	0.74 (0.18-3.01)		
	overdominant	***GG-AA***	107 (72.8)	153 (77.7)	1.00	0.299	472.5
		GA	40 (27.2)	44 (22.3)	0.77(0.47-1.26)		
	log-additive	***0,1,2***	147 (42.7)	197 (57.3)	0.78 (0.51-1.19)	0.255	472.3

Table S3. Analysis of correlation of densitometric results with age and BMI of patients in individual groups.

**Parameters**		**Control N=147**		**Osteopenia N=98**		**Osteoporosis N=197**	
**Parameter 1**	**Parameter 2**	**rho**	***p***	**rho**	***p***	**rho**	***p***
Age	BMI	0.56	<0.001	0.34	0.006	0.22	0.354
Age	L2L4 BMD	0.11	>0.999	-0.11	>0.999	-0.23	0.354
Age	L2L4 YA	0.13	>0.999	-0.11	>0.999	-0.21	0.354
Age	L2L4AM	0.47	0.003	0.53	<0.001	0.41	0.002
Age	T-score	0.15	>0.999	-0.14	>0.999	-0.23	0.307
Age	Z-score	0.60	0.003	0.51	<0.001	0.30	0.138
BMI	L2L4 BMD	0.25	0.462	0.02	>0.999	0.16	0.666
BMI	L2L4 YA	0.24	0.462	0.02	>0.999	0.17	0.666
BMI	L2L4AM	0.14	>0.999	-0.09	>0.999	-0.07	0.953
BMI	T-score	0.32	0.095	0.04	>0.999	0.14	0.666
BMI	Z-score	0.14	>0.999	-0.09	>0.999	-0.09	0.953

p - Spearman's rho with Holm's correction.
